# Adverse perinatal outcomes associated with prenatal exposure to protease-inhibitor-based versus non-nucleoside reverse transcriptase inhibitor-based antiretroviral combinations in pregnant women with HIV infection: a systematic review and meta-analysis

**DOI:** 10.1186/s12884-023-05347-5

**Published:** 2023-01-30

**Authors:** Laura Saint-Lary, Justine Benevent, Christine Damase-Michel, Christophe Vayssière, Valériane Leroy, Agnès Sommet

**Affiliations:** 1 Inserm U1295, CERPOP (Center for Epidemiology and Research in POPulation Health), Team SPHERE (Study of Perinatal, Paediatric and Adolescent Health: Epidemiological Research and Evaluation), Université Paul Sabatier Toulouse 3, 37 Allées Jules Guesde, 31000 Toulouse, France; 2 Service de Pharmacologie Clinique, CHU de Toulouse, Université Toulouse 3, Toulouse, France; 3 Service de Gynécologie-Obstétrique, CHU de Toulouse Purpan, Toulouse, France

**Keywords:** Antiretroviral therapy, HIV, Perinatal outcomes, Pregnancy, Protease inhibitor, Systematic review

## Abstract

**Background:**

About 1.3 million pregnant women lived with HIV and were eligible to receive antiretroviral therapy (ART) worldwide in 2021. The World Health Organization recommends protease inhibitors (PI)-based regimen as second or third-line during pregnancy. With remaining pregnant women exposed to PIs, there is still an interest to assess whether this treatment affects perinatal outcomes. Adverse perinatal outcomes after prenatal exposure to PI-based ART remain conflicting: some studies report an increased risk of preterm birth (PTB) and low-birth-weight (LBW), while others do not find these results. We assessed adverse perinatal outcomes associated with prenatal exposure to PI-based compared with non-nucleoside reverse transcriptase (NNRTI)-based ART.

**Methods:**

We performed a systematic review searching PubMed, Reprotox, Clinical Trial Registry (clinicaltrials.gov) and abstracts of HIV conferences between 01/01/2002 and 29/10/2021. We used Oxford and Newcastle-Ottawa scales to assess the methodological quality. Studied perinatal outcomes were spontaneous abortion, stillbirth, congenital abnormalities, PTB (< 37 weeks of gestation), very preterm birth (VPTB, < 32 weeks of gestation), LBW (< 2500 grs), very low-birth-weight (VLBW, < 1500 g), small for gestational age (SGA) and very small for gestational age (VSGA). The association between prenatal exposure to PI-based compared to NNRTI-based ART was measured for each adverse perinatal outcome using random-effect meta-analysis to estimate pooled relative risks (RR) and their corresponding 95% confidence intervals (CI). Pre-specified analyses were stratified according to country income and study quality assessment, and summarized when homogeneous.

**Results:**

Out of the 49,171 citations identified, our systematic review included 32 published studies, assessing 45,427 pregnant women. There was no significant association between prenatal exposure to PIs compared to NNRTIs for VPTB, LBW, SGA, stillbirth, and congenital abnormalities. However, it was inconclusive for PTB, and PI-based ART is significantly associated with an increased risk of VSGA (sRR 1.41 [1.08-1.84]; I^2^ = 0%) compared to NNRTIs.

**Conclusions:**

We did not report any significant association between prenatal exposure to PIs vs NNRTIs-based regimens for most of the adverse perinatal outcomes, except for VSGA significantly increased (+ 41%). The evaluation of antiretroviral exposure on pregnancy outcomes remains crucial to fully assess the benefice-risk balance, when prescribing ART in women of reproductive potential with HIV.

**PROSPERO number:**

CRD42022306896.

## Background

In 2021, 38 million people lived with HIV worldwide, of whom 1.3 million were pregnant women, and mostly in sub-Saharan Africa [1]. Maternal HIV infection among antiretroviral drug-naïve women is associated with a significantly increased risk of adverse perinatal outcomes, such as prematurity, low-birth-weight, small for gestational age and stillbirth [2]. Antiretroviral therapy (ART) during pregnancy has demonstrated a clear benefit for maternal health, and prevent the risk of HIV mother-to-child-transmission (MTCT) [3]. Since 2015, ART initiation is recommended in all people living with HIV, including pregnant women [4]. As a result, in 2021, 1.05 million pregnant women had access to antiretroviral drug regimen for their own health and prevention of MTCT (PMTCT), with 90% living in sub-Saharan Africa [1, 5]. In 2016, the World Health Organization (WHO) recommended a non-nucleoside-reverse-transcriptase-inhibitor (NNRTI)-based combination as the preferred first-line regimen, while protease inhibitor (PI)-based combinations were recommended as second or third-line regimen mainly due to incomplete information regarding its risk when used during pregnancy [6]. In 2019, the WHO recommended in all adults living with HIV a transition to dolutegravir (DTG)-based ART, despite a slightly higher but significant neural tube defect signal associated with DTG exposure in the pre-conception period compared to other antiretroviral combinations [7]. After all, a DTG-based combination was recommended in all adults, including pregnant women, as the preferred first-line regimen due to improved efficacy, better tolerability and durability compared to all previous ART regimens [3, 8]. Despite the beneficial effects of antiretroviral drugs during pregnancy on both maternal health and PMTCT, their use raises concerns on their potential embryo-foeto-toxicity. It remains crucial to fully assess their associated perinatal outcomes to optimise ART strategies in pregnant women worldwide, but more particularly in sub-Saharan Africa, where both maternal HIV prevalence and rates of adverse perinatal outcomes are high [5]. Several studies have reported an increased risk of adverse perinatal outcomes after prenatal exposure to antiretroviral combinations, depending on the antiretroviral drug classes used [9–16]. PI-regimens still remain an important alternative option for pregnant women in 2022 that still need to be fully understood, due to conflicting results. Indeed, several studies have reported an association between PI-based combinations and preterm birth, while other studies have not found similar results [12, 15–18]. Therefore, we conducted a systematic review and meta-analysis aimed to assess the risk of adverse perinatal outcomes associated with PI-based combination use during pregnancy compared to NNRTI-based combination.

## Methods

### Search strategy and selection criteria

We did a systematic review and meta-analysis according to the Preferred Reporting Items for Systematic review and Meta-Analysis (PRISMA) guidelines [19]. The protocol of this review was registered in PROSPERO, the International prospective register of systematic reviews (CRD42022306896). The bibliographic research was based on both published and unpublished studies from 01/01/2002 to 29/10/2021 relative to adverse perinatal outcomes in HIV women who received antiretroviral combination during pregnancy.

Searches were conducted on four electronic scientific literature databases: PubMed, Reprotox, Clinical Trial registry (clinicaltrials.gov) and the abstracts from HIV conferences (Conference on Retroviruses and Opportunists Infections, International AIDS Society, European AIDS Clinical Society, British HIV Association and International Workshop on HIV Pediatrics). We used the keywords and MeSH terms presented in the Table 1.

**Table 1:** Keywords and MeSH terms used in bibliographical researches

Data sources	Keywords and MeSH terms
Electronic scientific literature database (Pubmed)	("pregnancy outcome"[MeSH Terms] OR ("pregnancy"[All Fields] AND "outcome"[All Fields]) OR "pregnancy outcome"[All Fields] OR ("pregnancy"[All Fields] AND "outcomes" [All Fields]) OR "pregnancy outcomes"[All Fields]) AND ("hiv"[MeSH Terms] OR "hiv"[All Fields]) AND (antiretroviral [All Fields] OR cART [All Fields])
Electronic scientific literature databases (Reprotox, Clinical Trial registry (clinicaltrials.gov)) and abstracts from HIV conferences	“3TC, ABC, AZT, ZDV, d4T, TDF, FTC, NRTI, NNRTI, nucleoside, nucleotide, protease, DLV, EFV, ETR, NVP, APV, ATV, DRV, IDV, LPV, RTV, NFV, TPV, T-20, MVC, Atripla, lamivudine, abacavir, zidovudine, stavudine, zalcitabine, didanosine, emtricitabine, epzicom, kivexa, Trizivir, Combivir, Truvada, delavirdine, efavirenz, nevirapine, amprenavir, fosamprenavir, atazanavir darunavir, indinavir, lopinavir, ritonavir, saquinavir, tipranavir, enfurvitide, maraviroc, raltegravir, tenofovir, breast, mother, infant, baby, pregnant, pregnancy, perinatal, postnatal, feeding, breastfeeding, vertical, mtct, pmtct, “when to start” OR timing OR (“early” AND “initia*”)”

### Inclusion and exclusion criteria

To be eligible, studies must document population (pregnant women with documented perinatal outcomes) and exposure (antiretroviral combination based either on PI or NNRTI, initiated before or during pregnancy). We included all randomised controlled clinical trials, prospective and retrospective cohort studies using a comparative study design. Studies not eligible were those off-topic, not specifying the antiretroviral combination used, those where numbers of adverse perinatal outcomes according to antiretroviral combination were not detailed, those not comparing PI-based versus NNRTI-based antiretroviral combinations, studies with only one type of inhibitor used, and those where integral text was not available. For abstracts, we limited our search to studies in the English or French language. No restriction was applied to geographic area. Study investigators were contacted when the manuscript content was insufficient.

### Outcomes

We studied the following adverse perinatal outcomes based on WHO definitions: preterm birth (PTB, < 37 weeks of gestation) [20], very preterm birth (VPTB, < 32 weeks of gestation), low birth weight (LBW, < 2500 g) [21], very low birth weight (VLBW, < 1500 g), small for gestational age (SGA, birthweight < 10^th^ centile for gestational age) [22], very small for gestational age (VSGA, birthweight < 3^th^ percentile for gestational age), stillbirth (foetus born with no sign of life after 28 weeks of gestation) [23], congenital abnormalities (alteration in embryonal development) [24] and spontaneous abortion (< 22 weeks of gestation) [25]. Gestational age was estimated based on the last menstrual period and confirmed by ultrasound when available.

### Exposure variable

Pregnant women were considered exposed to antiretroviral combination if they started antiretroviral treatment before or during pregnancy, and continued at least until delivery. Antiretroviral combination was defined by at least three drugs: namely two nucleoside reverse transcriptase inhibitors (NRTI) associated with a PI (lopinavir/ritonavir, atazanavir/ritonavir, darunavir/ritonavir, fosamprenavir, saquinavir and nelfinavir) or a NNRTI (efavirenz or nevirapine). We categorised the exposure into three different periods: pre-conception, early pregnancy (first trimester) and late pregnancy (second and third trimester).

### Data extraction and quality analysis

Two investigators, LSL and VL independently reviewed and identified the relevant citations. LSL performed data extraction including description of the studies, their populations, the adverse perinatal outcomes according to antiretroviral combination, and scores of methodological qualities. We used two scales for methodological quality assessment according to study design: the Oxford scale [26] for clinical trials and the Newcastle–Ottawa scale [27] for cohort studies. Methodological quality assessment was conducted by two investigators independently (LSL and JB) and any discordance was resolved by discussion with AS. Studies with an Oxford score lower than three [26] or a Newcastle–Ottawa score lower than four [27] were considered as low methodological quality and were excluded.

### Statistical analysis

We first described the characteristics of the studies included. Then, we extracted data from individual studies to generate a relative risk (RR) of prenatal exposure to a PI-based combination compared to those with an NNRTI-based combination for each adverse perinatal outcome. We performed a meta-analysis when more than one study reported the same outcome, using a random effects model to estimate a weighted summary RR and corresponding 95% confidence intervals for each outcome [28, 29]. All pre-specified analyses were stratified according to country income: High-Income Countries (HIC) and Low-to-Middle-Income Countries (LMIC). We investigated between-study heterogeneity by reporting forest plots and using the I^2^ statistic, with a p-value significance of 0.10 (I² <0.10) [29]. The pooled summarised RR (sRR) was presented only when both LMIC and HIC RRs were consistent (I^2^ < 0.10). We searched publication bias using funnel-plot and asymmetric Egger tests [28]. We conducted sensitivity analyses excluding outlier studies by graphical research, and then including only studies with high score of methodological quality (Oxford score higher than five [26] and Newcastle–Ottawa score higher than seven [27]). For all analyses, we defined significance at an alpha level of 0.05 (p-value <0.05), except for heterogeneity analyses. Statistical analyses were performed using STATA (14.2).

## Results

### Study and population characteristics

**Fig. 1:**
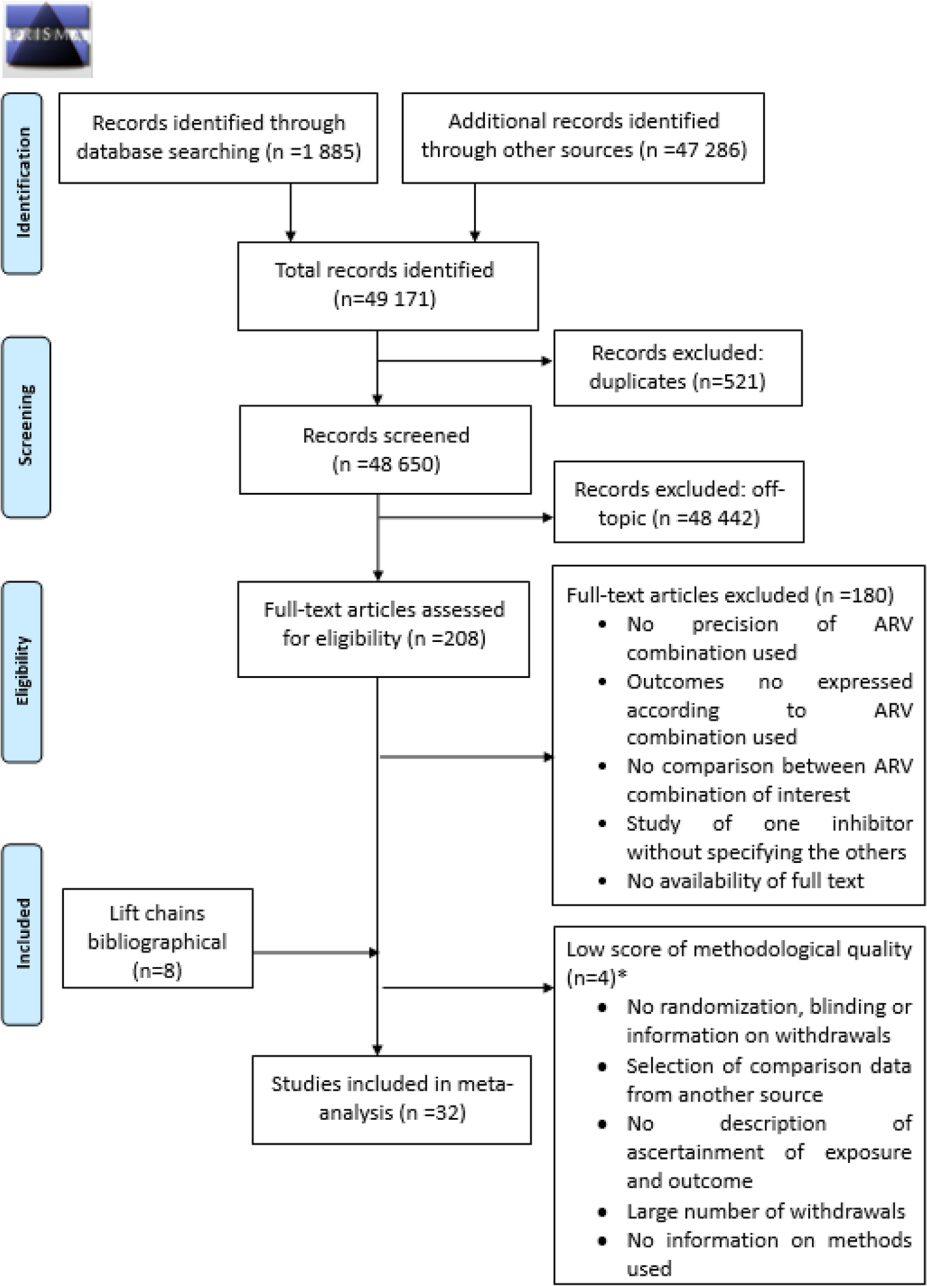
Flow-chart of study selection process according to PRISMA guidelines *Three studies had low score of methodological quality [30–32]. We could not assess methodological quality of one unpublished cohort study [33]: we considered this study of low methodological quality

Our search identified 49,171 citations: 1,885 published studies and 47,286 unpublished studies. Initial screening was from title and abstracts of studies in 48,650 records, after exclusion of duplicates. Overall, 208 full-text articles were selected for a complete reading. Finally, after excluding four studies of low methodological quality (two clinical trials [30, 31] and two cohort studies [32, 33]), 32 studies were retained for systematic review and meta-analysis (Fig. 1). These studies were published between 2002 and 2021, and included 45,427 pregnant women from 27 countries. Nineteen (59%) studies were conducted in HIC and thirteen (41%) in LMIC. Only one randomised controlled trial (3.1%) was selected, those remaining being cohort studies. Overall, thirteen studies (40.6%) had a high score of methodological quality (Table 2). Study sample size varied from 75 to 7,009 pregnant women, maternal age from 26 to 33 years and median CD4 + from 154 to 638 cells/mm^3^ (Table 3).

**Table 2:** Summary characteristics of the 32 selected studies and their methodological quality score

Authors and publication date	Countries	Study period	Study design	Inclusion criteria	Exclusion criteria	Jadad scale	Newcastle–Ottawa scale
Delicio et al. (2018) [34]	Brazil	2000–2015	Single-site cohort	Pregnant women HIV infected and new-borns seen at the Obstetrics Clinic; women with or without health insurance	Not mentioned	NA	8/9
Townsend et al. (2006) [35]	UK, Ireland	1990—2003	Multi-site cohort (NSHPC)	Live birth or stillbirth in women diagnosed before delivery; Delivery reported to the NSHPC before 2004	No information about antiretroviral combination and exposure period	NA	5/9
Malaba et al. (2017) [36]	South Africa	2013–2015	Prospective single-site cohort	Women attending their first antenatal care visit; Women with antiretroviral drugs eligibility; HIV-infected women conceiving while on ART continued their current regimen throughout pregnancy; regimens included PIs or NNRTIs; live singleton birth	Women not eligible for antiretroviral drugs at their first ANC visit (receiving zidovudine prophylaxis)	NA	8/9
Phiri et al. (2015) [37]	United States	1994–2009	National cohort (from TennCare)	Pregnant women enrolled in Tennessee Medicaid (TennCare); evidence of HIV infection; singleton live birth enrolled in Medicaid	Not mentioned	NA	6/9
Brogly et al. (2010) [38]	United states	1993–2000	Multi-site cohort	Children born to HIV-infected women and enrolled before one year of age	Not mentioned	NA	7/9
Prieto et al. (2014) [39]	Spain	2000–2009	Multi-site prospective cohort	HIV-infected mother-infants pairs with a definitive outcome through December 31, 2009	Stillbirth, termination of pregnancy, minor congenital anomalies	NA	7/9
Floridia et al. (2013) [40]	Italy	2001–2011	Prospective national cohort	Pregnant women with HIV, in routine clinical care, exposed to ARV	Miscarriage, voluntary termination of pregnancy for psychosocial reasons, and late HIV diagnosis (no ARV treatment before delivery or maternal diagnosis of HIV after delivery)	NA	5/9
van der Merwe et al. (2011) [41]	South Africa	2004—2007	Retrospective multi-site cohort	HIV pregnant women attending in clinics of the study; Singleton pregnancy; Women with CD4 count ≤ 250cells/mm^3^	Not have given consent	NA	5/9
Watts et al. (2013) [18]	United States	2007—2010	Prospective multi-site cohort	Pregnant women; Singleton pregnancy	No information about antiretroviral combination, obstetric data or gestational age	NA	6/9
Zash et al (2017) [42]	Botswana	2014—2016	Prospective multi-site cohort	Live birth or stillbirth in women who delivered at government maternity wards in Botswana	Births that occurred before arrival at the hospital or before 24 weeks	NA	7/9
Williams et al. (2015) [43]	United States	2007–2012	Prospective multi-site cohort	HIV-infected pregnant women and their children enrolled in the SMARTT study; documented antiretroviral drugs during pregnancy and pregnancy outcome	Not mentioned	NA	7/9
Schulte et al. (2007) [10]	United States	1989—2004	Prospective multi-site cohort (PSD)	Information about birth weight, gestational age and HIV status in the 30 first day of life	Not mentioned	NA	5/9
Patel et al (2005) [44]	Europe	1986—2003	Prospective multi-site cohort (ECS)	Not mentioned	Not mentioned	NA	5/9
Hu et al. (2019) [45]	China	2009–2018	Prospective single-site cohort	Pregnant women HIV-infected, exposed to antiretroviral drugs, reported to the IPMTCT system	HIV-infected pregnant women who elected to terminate their pregnancy	NA	7/9
Grosch-Woerner et al. (2008) [46]	Germany, Austria	1995—2001	Prospective multi-site cohort	HIV pregnant women identified from one center in Germany or Austria between 1995 and 2011	No information about antiretroviral exposure	NA	6/9
Cotter et al. (2006) [47]	United States	1990—2002	Prospective multi-site cohort	Singleton pregnancy; Women who received prenatal care in the hospital	Not mentioned	NA	4/9
Carceller et al. (2009) [48]	Canada	1997—2005	Retrospective single-site cohort	HIV pregnant women who received HAART; Women who received prenatal care and delivered in Sainte-Justine hospital	Women who did not received HAART; Women who received only one or two antiretroviral drugs	NA	4/9
Natureeba et al. (2014) [49]	Uganda	2009–2013	Open-label, single-site, randomized controlled trial	Women ≥ 16 years, infected with HIV-1 at any CD4 cell count, lived within 30 km of the study site, and had a pregnancy between 12–28 weeks gestation	Women who had ever received highly active combination ART or single dose nevirapine or other abbreviated monotherapy or dual therapy in the last 24 months; Women who had prior dose-limited toxicity to TMP-SMX within 14 days, active tuberculosis or other WHO stage 4 diseases, cardiac disease, or abnormal screening laboratory values including, hemoglobin 225 U/L, AST > 225 U/L, total bilirubin ≥ 2.5 times the upper limit of normal, and creatinine ≥ 1.8 times the upper limit of normal	3/5	-
Szyld et al (2006) [50]	Argentina, Bahamas, Brazil, Mexico	2002—2005	Prospective multi-site cohort (NISDI)	First enrolment in NISDI of HIV pregnant women; Women who received at least antiretroviral drugs for 28 days during pregnancy; Live and singleton birth; Information about birth weight and gestational age before 1 March 2005	Women who were still pregnant on 01 March 2005; Stillbirth; Miscarriage	NA	6/9
Joao et al (2010) [51]	Argentina, Brazil	2002—2007	Prospective multi-site cohort	Women enrolled in the NISDI for the first time; Singleton infant ≥ 20 weeks (live birth or stillbirth)	No follow-up during pregnancy; Second enrolment; No singleton pregnancy	NA	6/9
EPPICC Study Group (2019) [52]	East and West Europe	2008—2014	Multi-site cohort (EPPICC)	Singleton live-birth; No antiretroviral exposure before pregnancy; Antiretroviral exposure during pregnancy (only one antiretroviral combination)	Antiretroviral exposure < 2 weeks; Interruption or change of antiretroviral combination; No information about gestational age	NA	6/9
Snijdewind et al. (2018) [53]	Netherlands	1997—2015	Retrospective multi-site cohort (ATHENA)	Singleton live-born ≥ 24 weeks; Women who received antiretroviral combination (at least three drugs) and prenatal care in one of 26 centers in the Netherlands	Stillbirth; Miscarriage; Induced abortion; No information about pregnancy outcome	NA	5/9
Kreitchmann et al. (2014) [54]	Latin America, Caribbean	2002—2012	Prospective multi-site cohort	HIV pregnant women; First enrolment in the study	Not mentioned	NA	5/9
Floridia et al. (2020) [55]	Italy	2001–2011	Prospective national cohort	Live birth with documented outcome; date of last menstrual period after January 1 2008; use in pregnancy of three-drug regimens composed of a NRTI backbone plus a PI, a NNRTI or an ISTI	No treatment; monotherapy or dual therapy during pregnancy; triple NRTI regimens; switching to a different drug class during pregnancy; concomitant use of two classes among PI, NNRTI, and ISTI; start of antiretroviral treatment after 32 weeks of gestation; use of zidovudine, didanosine, stavudine, saquinavir, amprenavir, nelfinavir or tipranavir	NA	8/9
Ejigu et al. (2019) [56]	Ethiopia	2010–2016	Retrospective multi-site cohort	Antiretroviral exposed pregnancies; women attending prenatal care follow-up	Missing information about ART regimen, gestational age at birth and birth weight; ART regimen changed during pregnancy; exposure to ART for less than 2 weeks; abortions or multiple births	NA	7/9
Machado et al. (2008) [57]	Brazil	1996—2006	Prospective single-site cohort	HIV pregnant women	Miscarriage	NA	5/9
Stringer et al. (2018) [58]	Botswana, Kenya, Malawi, South Africa, Uganda, Zambia, Zimbabwe, Brazil, Haiti, India, Peru, Thailand, United States	2005–2015	Cohort based on three randomized clinical trials	Women included in three randomized clinical trials of HIV prevention or treatment conducted by the US National Institutes of Health, Division of AIDS Clinical Trials Networks, and the National Institute of Allergy and Infectious Diseases in resource-limited settings; Women with documented last menstrual period and pregnancy outcome; antiretroviral drugs exposure prior to conception; singleton birth	Not mentioned	NA	6/9
Patel et al (2010) [59]	United States, Puerto Rico	2002—2008	Prospective multi-site cohort	HIV pregnant women who were ≥ 13 years old; Singleton pregnancy; First enrolment in the study; Women with estimated date of conception at least 10 months before 5 March 2008; Women with at least one CD4 + count obtained during pregnancy; Women who did not received antiretroviral drugs at conception or within 6 months prior to conception	No exposure to antiretroviral combination during pregnancy	NA	7/9
Favarato et al. (2018) [60]	UK, Ireland	2007–2015	Prospective multi-site cohort (NSHPC)	Pregnancies with documented gestational age resulting in a singleton live birth; women diagnosed with HIV before delivery and reported to the NSHPC by March 2016	Not mentioned	NA	7/9
Aaron et al. (2012) [61]	United States	2000–2011	Prospective single-site cohort	HIV pregnant women aged 17 and more; First pregnancy and first infant delivered in twin gestations	Abortion or miscarriage; switched prenatal providers; incarceration during the index pregnancy	NA	8/9
Favarato et al. (2019) [62]	UK, Ireland	2007–2015	Prospective multi-site cohort (NSHPC)	Pregnancies in HIV women resulting in a live birth or stillbirth at ≥ 24-week gestation reported in NSHPC by March 2017	Not mentioned	NA	7/9
Bellón Cano et al. (2004) [63]	Spain	1997—2000	Prospective multi-site cohort	HIV pregnant women who received antiretroviral combination during pregnancy	Not mentioned	NA	5/9

*ALT* Alanine aminotransferase, *AST* Aspartame aminotransferase, *ATHENA* *AIDS* Therapy Evaluation in the Netherlands, *ECS* European Collaborative Study, *HAART* Highly Active Antiretroviral Therapy, *HIV* Human Immunodeficiency Virus, *IPMTCT* Integrated Prevention of Mother To Child Transmission, *EPPICC* European Pregnancy and Paediatric HIV Cohort Collaboration, *NA* Not available, *NSHPC* National Study of HIV in Pregnancy and Childhood, *NISDI* NICHD (National Institute of Child Health & Human Development) International Site Development Initiative, *PSD* Pediatric Spectrum of HIV Disease, *SMARTT* Surveillance Monitoring of ART Toxicities, *TS* Trimethoprim-sulfamethoxazole, *WHO* World Health Organisation

**Table 3:** Description of the study population and summary results for studies included in the systematic review

Authors and publication date	Sample size	Characteristics of subjects	Antiretroviral combination exposure	Exposure period to antiretroviral combination	Outcomes	Results (Percentages and CI 95%)	Relative risk and CI 95%
Delicio et al. (2018) [34]	*N* = 801	Median age 28 years [13-46] CD4 + median 444cell/ml [3–1915] Caucasian ethnic 61%	PI = 603 NNRTI = 141	Before conception *n* = 217	Preterm birth	PI = 130/579 (22.5 [19.1–25.9]) NNRTI = 25/141 (17.7 [11.4–24.0])	1.27 [0.86–1.86]
					LBW	PI = 135/591 (22.8 [19.5–26.2]) NNRTI = 28/142 (19.7 [13.2–26.3])	1.16 [0.81–1.67]
					SGA	PI = 118/582 (20.3 [17.0–23.5]) NNRTI = 14/141 (9.9 [5.0–14.9])	2.04 [1.21–3.44]
Townsend et al (2006) [35]	*N* = 3 147	Median age 29 years [16-44] Median CD4 + ND Caucasian ethnic 18.7%, African ethnic 73.6%	PI *n* = 205 NNRTI *n* = 291	First trimester *n* = 541, whose HAART *n* = 496	Congenital abnormalities	PI = 7/205 (3.4 [0.9–5.9]) NNRTI = 12/291(4.1[1.8–6.4])	0.83 [0.33–2.07]
Malaba et al. (2017) [36]	*N* = 1 494	Median age 29 years [26-34] CD4 count (cell/µl) ≤ 350 43. > 350 54	PI = 33 NNRTI = 1245	Before conception *n* = 572	Preterm birth	PI = 10/29 (34.5 [17.2–51.8]) NNRTI = 217/961 (22.6 [19.9–25.2])	1.53 [0.91–2.56]
					VPT	PI = 0/29 (0 [0–0]) NNRTI = 23/961 (2.4 [1.4–3.4])	0.68 [0.04–1.96]
					LBW	PI = 5/29 (17.2 [3.5–31.0]) NNRTI = 139/961 (14.5 [12.2–16.7])	1.19 [0.53–2.69]
					VLBW	PI = 0/29 (0 [0–0]) NNRTI = 20/961 (2.1 [1.2–3.0])	0.78 [0.05–12.63]
					SGA	PI = 3/29 (10.3 [0.0–21.4]) NNRTI = 111/961 (11.6 [9.5–13.6])	0.90 [0.30–2.65]
Phiri et al. (2015) [37]	*N* = 3 228	Median age 26 years [14-43] Caucasian ethnic 19%, African ethnic 81%	PI = 222 NNRTI = 78	ND	Preterm birth	PI = 41/222 (18.5 [13.4–23.6]) NNRTI = 14/78 (17.9 [9.4–26.5])	1.03 [0.59–1.78]
					VPT	PI = 9/222 (4.1 [1.5–6.6]) NNRTI = 3/78 (3.8 [0.0–8.1])	1.05 [0.29–3.80]
					LBW	PI = 43/222 (19.4 [14.2–24.6]) NNRTI = 25/78 (32.1 [21.7–42.4])	0.60 [0.40–0.92]
					VLBW	PI = 6/222 (2.7 [0.6–4.8]) NNRTI = 1/78 (1.3 [0.0–3.8])	2.11 [0.26–17.24]
					SGA	PI = 55/222 (24.8 [19.1–30.5]) NNRTI = 28/78 (35.9 [25.3–46.5])	0.69 [0.47–1.00]
					Stillbirth	PI = 11/637 (1.7 [0.7–2.7]) NNRTI = 0/84 (0.0 [0.0–0.0])	3.06 [0.18–51.53]
Brogly et al. (2010) [38]	*N* = 2 202	Age < 20 years 6. 20–34 years 71. > 35 years 13 Non-Hispanic ethnic 67%, Hispanic ethnic 30%, Other ethnic 1%	PI = 353 NNRTI = 142	ND	Congenital abnormalities	PI = 23/353 (6.5 [3.9–9.1]) NNRTI = 8/142 (5.6 [1.8–9.4])	1.16 [0.53–2.52]
Prieto et al. (2014) [39]	*N* = 898	CD4 count at enrolment (cell/µl): > 500 30%, 200–500 28%, < 200 8% Caucasian ethnic 72%, Sub-Saharan ethnic 16%, Latino American ethnic 11%, other ethnic 2%	PI = 476 NNRTI = 233	First trimester *n* = 329 Second/third trimester *n* = 488	Congenital abnormalities	PI = 31/476 (6.5 [4.3–8.7]) NNRTI = 14/233 (6.0 [3.0–9.1])	1.08 [0.59–2.00]
Floridia et al. (2013) [40]	*N* = 2 830	Median age 33 years [29-69] Median CD4 + count (cell/mm^3^) 420 [295–575] Caucasian ethnic 66%, African ethnic 29%, other ethnic 4%	PI = 349 NNRTI = 257	Before conception *n* = 1257 First trimester *n* = 1257	Congenital abnormalities	PI = 11/353 (3.1 [1.3–4.9]) NNRTI = 11/273 (4.0 51.7–6.4])	0.77 [0.34–1.76]
van der Merwe et al. (2011) [41]	*N* = 1 630	Mean age 30.3 years (sd 5.1) Median CD4 + 154 cell/mm^3^ (101–195) Ethnic group ND	PI *n* = 445 NNRTI *n* = 534	Before 28 weeks *n* = 533 (PI *n* = 155 and NNRTI *n* = 398) After 28 weeks *n* = 427 (PI *n* = 290 and NNRTI *n* = 137)	Preterm birth	PI = 15/421 (3.6 [1.8–5.3] NNRTI = 44/395 (11.1 [8.0–14.2])	0.32 [0.18- 0.57]
					VPT^□^	PI = 13/421 (3.1 [1.4–4.7]) NNRTI = 30/395 (7.6 [5.0–10.2])	0.41 [0.22–0.77]
					LBW	PI = 64/419 (15.3 [11.8–18.7]) NNRTI = 92/376 (24.5 [20.1–28.8])	0.62 [0.47–0.83]
					VLBW	PI = 7/419 (1.7 [0.4–2.9]) NNRTI = 3/376 (0.8 [0.0–1.7])	2.09 [0.55- 8.04]
					SGA	PI = 147/403 (36.5 [31.8–41.2]) NNRTI = 57/309 (18.4 [14.1–22.8])	1.98 [1.51–2.58]
Watts et al (2013) [18]	*N* = 2 218	Median age 27 years [23-32] CD4 + (cell/mm^3^) < 200 13, 200–500 46 Caucasian ethnic 28%, African ethnic 65%, other ethnic 7%	PI *n* = 1319 NNRTI *n* = 160	ND	Preterm birth	PI = 255/1319 (19.3 [17.2–21.5]) NNRTI = 27/160 (16.9 [11.1–22.7])	1.15 [0.80–1.64]
					SGA	PI = 99/1319 (7.5 [6.1- 8.9]) NNRTI = 11/160 (6.9 [3.0–10.8])	1.09 [0.60–1.99]
Zash et al (2017) [42]	*N* = 47 027	Mean age 26.9 years (sd 6.5) Median CD4 + (cell/mm^3^) 478 [363–600] for TDF-FTC-EFV group, 508 [388–685] for TDF-FTC-NVP group, 638 [492–735] for TDF-FTC-LPV-R group, 504 [408–625] for ZDV-3TC-NVP group, 609 [414–798] for ZDV-3TC-LPV-R group Ethnic group ND	IP *n* = 398 INNTI *n* = 4597	Before conception *n* = 4812 (PI *n* = 398 and NNRTI *n* = 4597) After conception *n* = 5780 (PI *n* = 406 and NNRTI *n* = 468)	Preterm birth	PI = 104/398 (26.1 [21.8–30.4]) NNRTI = 1012/4597 (22.0 [20.8–23.2])	1.19 [1.00–1.41]
					VPT	PI = 27/398 (6.8 [4.3–9.3]) NNRTI = 220/4597 (4.8 [4.2–5.4])	1.42 [0.96–2.09]
					SGA	PI = 98/398 (24.6 [20.4–28.9]) NNRTI = 993/4597 (21.6 [20.4–22.8])	1.14 [0.95–1.37]
					VSGA	PI = 52/398 (13.1 [9.8–16.4]) NNRTI = 437/4597 (9.5 [8.7–10.4])	1.37 [1.05–1.80]
					Stillbirth	PI = 16/398 (4.0 [2.1–5.9]) NNRTI = 164/4597 (3.6 [3.0–4.1])	1.13 [0.68–1.86]
Williams et al. (2015) [43]	*N* = 2580	Age ≥ 35 years 13 CD4 < 250cell/mm^3^ 14 Caucasian ethnic 27%, African ethnic 66%, other ethnic 1%	PI = 887 NNRTI = 214	First trimester *n* = 1219	Congenital abnormalities	PI = 75/887 (8.5 [6.6–10.3]) NNRTI = 13/214 (6.1 [2.9–9.3])	1.39 [0.79–2.46]
Schulte et al (2007) [10]	*N* = 11 231	Median age ND Median CD4 + ND African ethnic 61.6%	PI n = 782 Non PI *n* = 1781	ND	Preterm birth	PI = 132/782 (16.9 [14.3–19.5]) Non PI = 329/1781 (18.5 [16.7–20.3])	0.91 [0.76- 1.10]
					LBW	PI = 133/782 (17.0 [14.4–19.6]) Non PI = 330/1781 (18.5 [16.7–20.3])	0.92 [0.77–1.10]
Patel et al (2005) [44]	*N* = 3 740	Median age 28 years [10-45] Median CD4 + 420 cell/ml [0–2350] Caucasian ethnic 72.2%, African ethnic 20.9%, other ethnic 6.8%	PI *n* = 273 NNRTI *n* = 195	First trimester *n* = 789 (PI *n* = 273 and NNRTI *n* = 195) Second/third trimesters *n* = 1184	Congenital abnormalities	PI = 7/273 (2.6 [0.7–4.4]) NNRTI = 6/195 (3.1 [0.7–5.5])	0.83 [0.28–2.44]
Hu et al. (2019) [45]	*N* = 802	Mean age 29.8 years (sd 5) Last CD4 count (cell/µl) < 350 23, ≥ 350 37 Asian ethnic 91%, other ethnic 9%	PI = 220 NNRTI = 146	ND	Preterm birth	PI = 34/220 (15.5 [10.7–20.2]) NNRTI = 23/146 (15.8 [9.8–21.7])	0.98 [0.60–1.60]
Grosch-Woerner et al. (2008) [46]	*N* = 190	Median age 28 years [17-41] CD4 + (cell/mm^3^) < 200 12, 200–500 59 Caucasian ethnic 47%, African ethnic 36%, other ethnic 16%	PI *n* = 21 NNRTI *n* = 54	ND	Preterm birth^∆^	PI = 13/21 (61.9 [41.1–82.7]) NNRTI = 16/54 (29.6 [17.5–41.8])	2.09 [1.23–3.55]
					Congenital abnormalities	IP = 2/21 (9.5 [0.0–22.1]) INNTI = 1/54 (1.9 [0.0–5.4])	5.14 [0.49–53.76]
Cotter et al (2006) [47]	*N* = 1 337	Age < 18 years 2.7, 18–34 years 79.1, > 34 years 14.0 CD4 + (cell/mm^3^) < 200 22.5, 200–499 58.3 Caucasian ethnic 4.3%, African ethnic 47.6%, Hispanic ethnic 13.2%, other ethnic 35%	PI *n* = 134 Non PI *n* = 373	Before 10 weeks *n* = 122 (PI *n* = 15 and non PI *n* = 107) After 10 weeks *n* = 384 (PI *n* = 118 and NNRTI *n* = 266)	Preterm birth	PI = 49/134 (36.6 [28.4–44.7]) Non PI = 101/373 (27.1 [22.6–31.6])	1.35 [1.02- 1.78]
					VPT	PI = 3/134 (2.2 [0.0–4.7]) Non PI = 17/373 (4.6 [2.4–6.7])	0.49 [0.15–1.65]
					LBW	PI = 23/134 (17.2 [10.8–23.5]) Non PI = 59/373 (15.8 [12.1–19.5])	1.09 [0.70–1.68]
					VLBW	PI = 3/134 (2.2 [0.0–4.7]) Non PI = 19/373 (5.1 [2.9–7.3])	0.44 [0.13–1.46]
					Stillbirth	PI = 0/134 (0.0 [0.0- 0.0]) Non PI = 2/373 (0.5 [0.0–1.2])	0.55 [0.03–11.47]
Carceller et al (2009) [48]	*N* = 206	Median age ND Median CD4 + ND Ethnic group ND	PI *n* = 176 Non PI *n* = 40	First trimester *n* = 78 Second trimester *n* = 92 Third trimester *n* = 33	Preterm birth	PI = 19/171 (11.1 [6.4–15.8]) Non PI = 2/28 (7.1 [0.0–16.7])	1.56 [0.38- 6.32]
					SGA*	PI = 17/174 (9.8 [5.4–14.2]) Non PI = 3/29 (10.3 [0.0–21.4])	0.94 [0.30- 3.02]
Natureeba et al. (2014) [49]	*N* = 389	Mean age 29.5 ± 5.4 Median CD4 count (cell/mm3): for EFV group 374 [270–485], for LPV/r group 368 [282–506]	PI = 194 NNRTI = 195	Not mentioned	Prematurity	PI = 39/190 (20.5 [14.8–26.3]) NNRTI = 34/187 (18.2[12.7–23.7])	1.13 [0.75–1.71]
					Low birth weight	PI = 39/181 (21.5 [15.6–27.5]) NNRTI = 33/178 (18.5[12.8–24.2])	1.16 [0.77–1.76]
Szyld et al (2006) [50]	*N* = 803	Age < 20 years 6.3, 20–29 years 52.4, > 29 years 40.5 CD4 + (cell/mm^3^) < 200 12.9, 200–499 54.3 Ethnic group ND	PI *n* = 330 NNRTI *n* = 257	ND	Preterm birth	PI = 35/330 (10.6 [7.3–13.9]) NNRTI = 15/257 (5.8 [3.0–8.7])	1.82 [1.01- 3.25]
					LBW	PI = 55/330 (16.7 [12.6–20.7]) NNRTI = 19/257 (7.4 [4.2–10.6])	2.25 [1.37–3.70]
Joao et al (2010) [51]	*N* = 1 229	Age < 20 years 6.9, 20–29 years 54.8, > 29 years 38.3 CD4 + (cell/mm^3^) < 200 12.6, 200–499 54.6 Ethnic group ND	PI *n* = 511 NNRTI *n* = 305	First trimester *n* = 249 Second trimester *n* = 92 Third trimester *n* = 33	Congenital abnormalities	PI = 25/511 (4.9 [3.0–6.8]) NNRTI = 25/305 (8.2 [5.1–11.3])	0.60 [0.35–1.02]
EPPICC Study Group (2019) [52]	*N* = 7193	Median age 29 years [25-33] CD4 + count (cell/µl) < 200 14, 200–349 26, ≥ 350 59 Caucasian ethnic 59%, African ethnic 37%, other ethnic 3%	PI = 6492 NNRTI = 517	ND	Preterm birth	PI = 653/6492 (10.1 [9.3–10.8]) NNRTI = 51/517 (9.9 [7.3–12.4])	1.02 [0.78–1.34]
					SGA	PI = 729/6399 (11.4 [10.6–12.2]) NNRTI = 39/510 (7.6 [5.3–10.0])	1.49 [1.09–2.03]
Snijdewind et al (2018) [53]	*N* = 1 392	Median age 29.9 years [25.8–34.4] Median CD4 + 520cell/µl [374–700] Caucasian ethnic 20.7%, African ethnic 61.3%, other ethnic 18.0%	IP *n* = 928 INNTI *n* = 438	Before conception *n* = 550 (PI *n* = 269 and NNRTI *n* = 263) After conception *n* = 842 (PI *n* = 659 and NNRTI *n* = 175)	Preterm birth	PI = 104/928 (11.2 [9.2–13.2]) NNRTI = 58/438 (13.2 [10.1–16.4])	0.85 [0.63–1.14]
					VPT	PI = 23/928 (2.5 [1.5–3.5]) NNRTI = 15/438 (3.4 [1.7–5.1])	0.72 [0.38–1.37]
					LBW	PI = 114/928 (12.3 [10.2–14.4]) NNRTI = 55/438 (12.6 [9.5–15.7])	0.98 [0.72–1.32]
					VLBW	PI = 23/928 (2.5 [1.5–3.5]) NNRTI = 21/438 (4.8 [2.8–6.8])	0.52 [0.29- 0.92]
					SGA	PI = 215/928 (23.2 [20.5–25.9]) NNRTI = 105/438 (24.0 [20.0–28.0])	0.97 [0.79–1.18]
Kreitchmann et al. (2014) [54]	*N* = 1 563	Mean age 28.2 years (sd 5.9) CD4 + (cell/mm^3^) < 200 9.5, 200–499 40.1 Caucasian ethnic 58%, African ethnic 20.4%, other ethnic 21.6%	PI *n* = 907 Non PI *n* = 409	First trimester *n* = 367 (PI *n* = 192 and NNRTI *n* = 152) Third trimester *n* = 1432 (PI *n* = 888 and NNRTI *n* = 410)	Combined outcome	PI = 310/907 (34.2 [31.1–37.3]) Non PI = 126/409 (30.8 [26.3–35.3])	1.11 [0.94–1.32]
Floridia et al. (2020) [55]	*N* = 794	Median age 32 years [28-36] Median CD4 (count/mm^3^) 473 [328.5–664] Caucasian ethnic 48%, African ethnic 46%, other ethnic 6%	PI = 623 NNRTI = 122	Before conception *n* = 794	Preterm birth	PI = 99/622 (15.9 [13.0–18.8]) NNRTI = 23/121 (19.0 [12-26] )	0.84 [0.56–1.26]
					VPT	PI = 13/622 (2.1 [1.0–3.2]) NNRTI = 1/121 (0.8 [0.0–2.4])	2.53 [0.33–19.15]
					LBW	PI = 113/597 (18.9 [15.8–22.1]) NNRTI = 24/124 (19.4 [12.4–26.3])	0.98 [0.66–1.45]
					VLBW	PI = 16/597 (2.7 [1.4–4.0]) NNRTI = 4/124 (3.2 [0.1–6.3])	0.83 [0.28–2.44]
					SGA	PI = 68/564 (12.1 [9.4–14.7]) NNRTI = 7/107 (6.5 [1.9–11.2])	1.84 [0.87–3.90]
Ejigu et al. (2019) [56]	*N* = 2412	Median age 29 years [26-32] Median CD4 + (cell/mm^3^) 384 [256–534]	PI = 32 NNRTI = 1432	Before conception *n* = 826	Preterm birth	PI = 8/32 (25.0 [10-40] ) NNRTI = 255/1432 (17.8 [15.8–19.8])	1.40 [0.76–2.59]
					LBW	PI = 4/32 (12.5 [1-24]) NNRTI = 298/1432 (20.8 [18.7–22.9])	0.60 [0.24–1.51]
					SGA	PI = 8/32 (25.0 [10-40]) NNRTI = 481/1432 (33.6 [31.1–36.0])	0.74 [0.41–1.36]
Machado et al (2008) [57]	*N* = 899	Median age 28.7 years for group who received antiretroviral combination before conception / 26.7 years for group who received antiretroviral combination after conception Median CD4 + ND Ethnic group ND	PI *n* = 213 NNRTI *n* = 100	Before conception n = 99 After conception n = 205	Preterm birth	PI = 30/213 (14.1 [9.4–18.8]) NNRTI = 11/100 (11.0 [4.9–17.1])	1.28 [0.67–2.45]
					LBW	PI = 33/213 (15.5 [10.6–20.4]) NNRTI = 13/100 (13.0 [6.4–19.6])	1.19 [0.66–2.16]
Stringer et al. (2018) [58]	N = 359	Median age 29 years [26-33] Median CD4 count (cell/mm^3^) 431 [304–608]	PI = 118 NNRTI = 127	Before conception *n* = 253	Preterm birth	PI = 45/118 (38.1 [29.4–46.9]) NNRTI = 36/127(28.3 [20.5–36.2])	1.35 [0.94–1.93]
					Stillbirth	PI = 4/118 (3.4 [0.1–6.7]) NNRTI = 5/127 (3.9 [0.6–7.3])	0.86 [0.24–3.13]
					Spontaneous abortion	PI = 27/118 (22.9 [15.3–30.5]) NNRTI = 23/127 (18.1 [11.4–24.8])	1.26 [0.77–2.08]
Patel et al (2010) [59]	*N* = 1 196	Age 14–21 years 21, 22–25 years 24, 26–29 years 25, ≥ 30 years 30 CD4 + (cell/mm^3^) < 200 15, 200–499 55 Caucasian ethnic 11%, African ethnic 58%, Hispanic ethnic 30%	PI *n* = 558 Non PI *n* = 219	First trimester *n* = 153 (PI *n* = 117 and NNRTI *n* = 36) Second trimester *n* = 507 (PI *n* = 363 and NNRTI *n* = 144) Third trimester *n* = 117 (PI *n* = 78 and NNRTI *n* = 39)	Preterm birth	PI = 90/558 (16.1 [13.1–19.2]) NNRTI = 27/219 (12.3 [8.0–16.7]	1.31 [0.88–1.95]
					VPT	PI = 12/558 (2.2 [0.9–3.4]) NNRTI = 1/219 (0.5 [0.0–1.3])	4.71 [0.62–36.00]
Favarato et al. (2018) [60]	*N* = 6073	Median age 33 years [29-36] Median CD4 count (cell/mm^3^) 440 [311–596] African ethnic 73%	PI = 4184 NNRTI = 1889	ND	Preterm birth	PI = 460/4184 (11.0 [10.0–11.9]) NNRTI = 169/1889 (8.9 [7.7–10.2])	1.13 [1.04–1.45]
					^□^VPT	PI = 157/4184 (3.8 [3.2–4.3]) NNRTI = 71/1889 (3.8 [2.9–4.6])	1.00 [0.76–1.31]
					SGA	PI = 825/4184 (19.7 [18.5–20.9]) NNRTI = 338/1889 (17.9 [16.2–19.6])	1.10 [0.98–1.24]
					VPT	PI = 3/179 (1.7 [0.0–3.6]) NNRTI = 3/177 (1.7 [0.0–3.6])	0.99 [0.20–4.83]
					Combined outcome	PI = 34/184 (18.5 [12.9–24.1]) NNRTI = 32/183 (17.5 [12.0–23.0])	1.06 [0.68–1.64]
Aaron et al. (2012) [61]	N = 183	Mean age 28.0 (sd 6.2) First CD4 > 200 73.2 African American ethnic 74.7%, all other ethnic 25.3%	PI = 117 NNRTI = 39	Before conception *n* = 46 During pregnancy *n* = 183	SGA	PI = 39/117 (33.3 [24.8–41.9]) NNRTI = 7/39 (17.9 [5.9–30.0])	1.86 [0.91–3.81]
					VSGA	PI = 17/117 (14.5 [8.1–20.9]) NNRTI = 2/39 (5.1 [0.0–12.1])	2.83 [0.69–11.72]
Favarato et al. (2019) [62]	*N* = 10,434	Age < 28–36 years 73, > 36 years 27 CD4 + (cell/mm^3^) > 350 62, ≤ 350 32 Caucasian ethnic 19%, African ethnic 75%, Hispanic ethnic 3%, Asian ethnic 2%	PI = 4693 NNRTI = 2259	Before conception *n* = 5023	Stillbirth	PI = 41/4693 (0.9 [0.6–1.1]) NNRTI = 19/2259 (0.8 [0.5–1.2])	1.04 [0.60–1.79]
Bellón Cano et al (2004) [63]	*N* = 124	Median age ND CD4 + (cell/mm^3^) < 250 27, 250–500 51 Ethnic group ND	PI n = 72 NNRTI *n* = 52	ND	Preterm birth	PI = 13/74 (17.6 [8.9–26.2]) NNRTI = 5/52 (9.6 [1.6–17.6])	1.83 [0.69–4.81]
					LBW	PI = 18/74 (24.3 [14.5–34.1]) NNRTI = 9/52 (17.3 [7.0–27.6])	1.41 [0.69–2.88]
					VLBW	PI = 3/74 (4.1 [0.0 – 8.5]) NNRTI = 0/52 (0.0 [0.0 – 0.0])	4.95 [0.26–93.78]
					Congenital abnormalities	PI = 4/74 (5.4 [0.3–10.6]) NNRTI = 4/52 (7.7 [0.4–14.9])	0.70 [0.18–2.68]

*HAART* Highly Active Antiretroviral Therapy, *CI95* Confidence interval 95, *LBW* Low birth weight, *ND* not define, *NNRTI* Non-nucleoside reverse transcriptase inhibitor, *non PI* considered here like NNRTI, *PI* Protease inhibitor, *SGA* Small for gestational age, *VLBW* Very low birth weight, *VPT* Very preterm birth, *VSGA* Very small for gestational age

Combined outcome: at least one adverse pregnancy outcome; LBW < 2500 g; VLBW < 1500 g; SGA < 10^ième^ percentile of curves of reference growth, except * > 37 weeks and < 2500 g or < 37 weeks and -2sd weight; VSGA < 3^ième^ percentile of curves of reference growth; Preterm birth < 37 weeks, except ^∆^ < 36 weeks; VPT < 32 weeks, except ^□^ < 34 weeks

Out of the 32 studies, gestational age was estimated using ultrasound scan alone, last menstrual period alone or a combination of at least two methods, in 3.2% (1/31), 16.1% (5/31) and 67.7% (21/35) respectively. Information on gestational age estimation methods was not available for five studies (15.6%), reporting only congenital abnormalities [35, 38, 39, 43, 51]. The most common perinatal outcomes reported were PTB (22/32, 68.8%), LBW (13/32, 40.6%) and SGA (13/32, 40.6%) (Table 3).

### Preterm birth

**Fig. 2:**
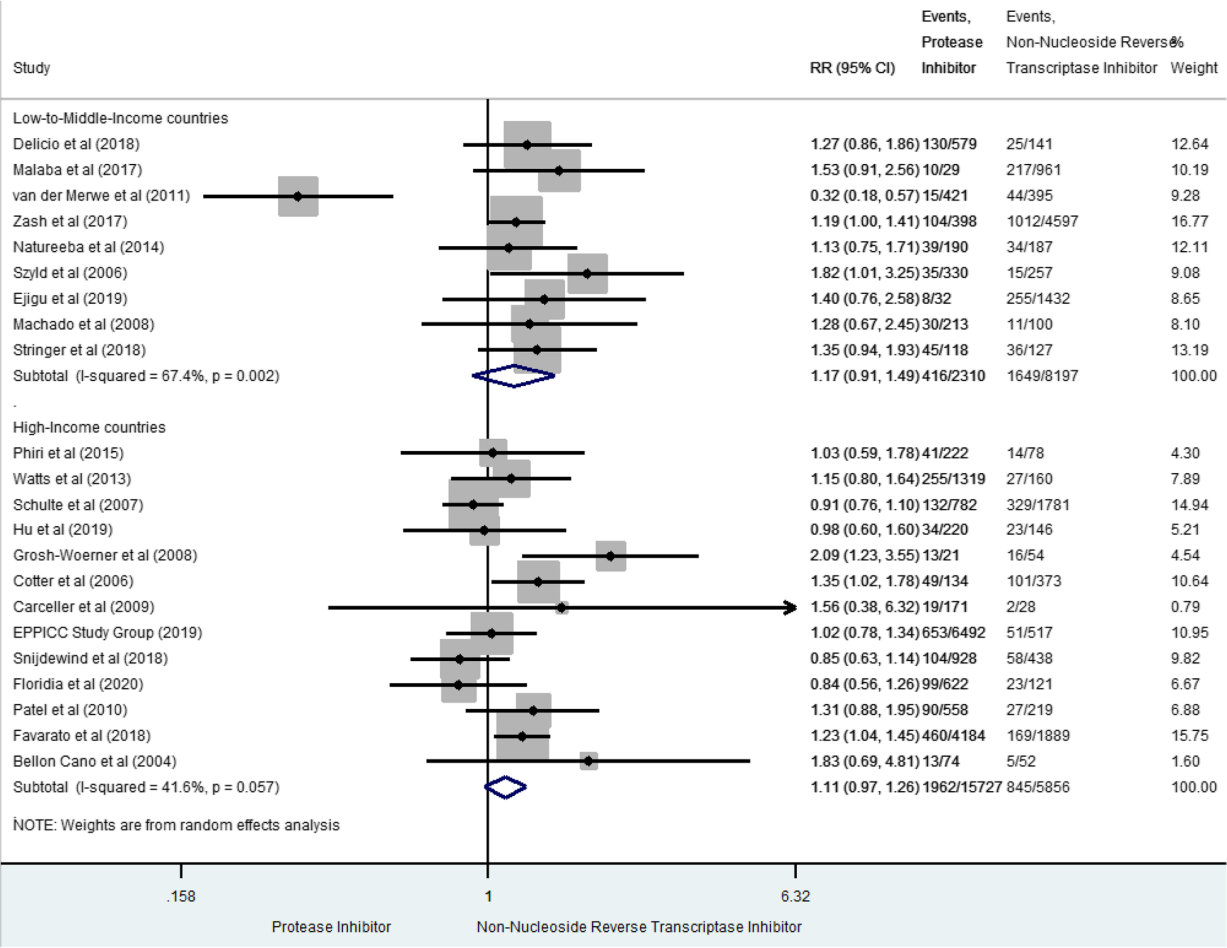
Forest-plot of preterm-birth risks in pregnant women receiving PI-based compared to NNRTI-based antiretroviral combination

PTB was reported in 4,872 cases in twenty-two studies (15.2% [14.8–15.6]) [10, 18, 36, 37, 41, 42, 45–50, 52, 53, 55–60, 63, 64] (Fig. 2). In both LMIC and HIC separately, prenatal exposure to PI-based combination was not significantly associated with PTB compared to NNRTI-based combination (RR 1.17, 95%CI 0.91–1.49 and RR 1.11, 95%CI 0.97–1.26, respectively), but between-study heterogeneity was significant (I^2^ 67.4% *p* = 0.002 and 41.6% *p* = 0.057 respectively). No global summary estimate was provided due to significant heterogeneity. More specifically, in LMIC, heterogeneity was only due to the van der Merwe study [41], the single outlier with opposite results compared to the eight others studies. When excluding this study, the RR became significant (RR 1.26, 95%CI 1.11–1.43) and homogeneous (I^2^ 0%, *p* = 0.882). When including only studies with high score of methodological quality in LMIC and HIC [36, 42, 45, 55, 56, 59, 60, 64], the results were also homogeneous with a global significant increased risk of PTB after prenatal exposure to PI-based combination, compared to NNRTI-based combination (sRR 1.20 [1.08–1.32], I^2^ 0% *p* = 0.653).

### Very preterm birth

**Fig. 3:**
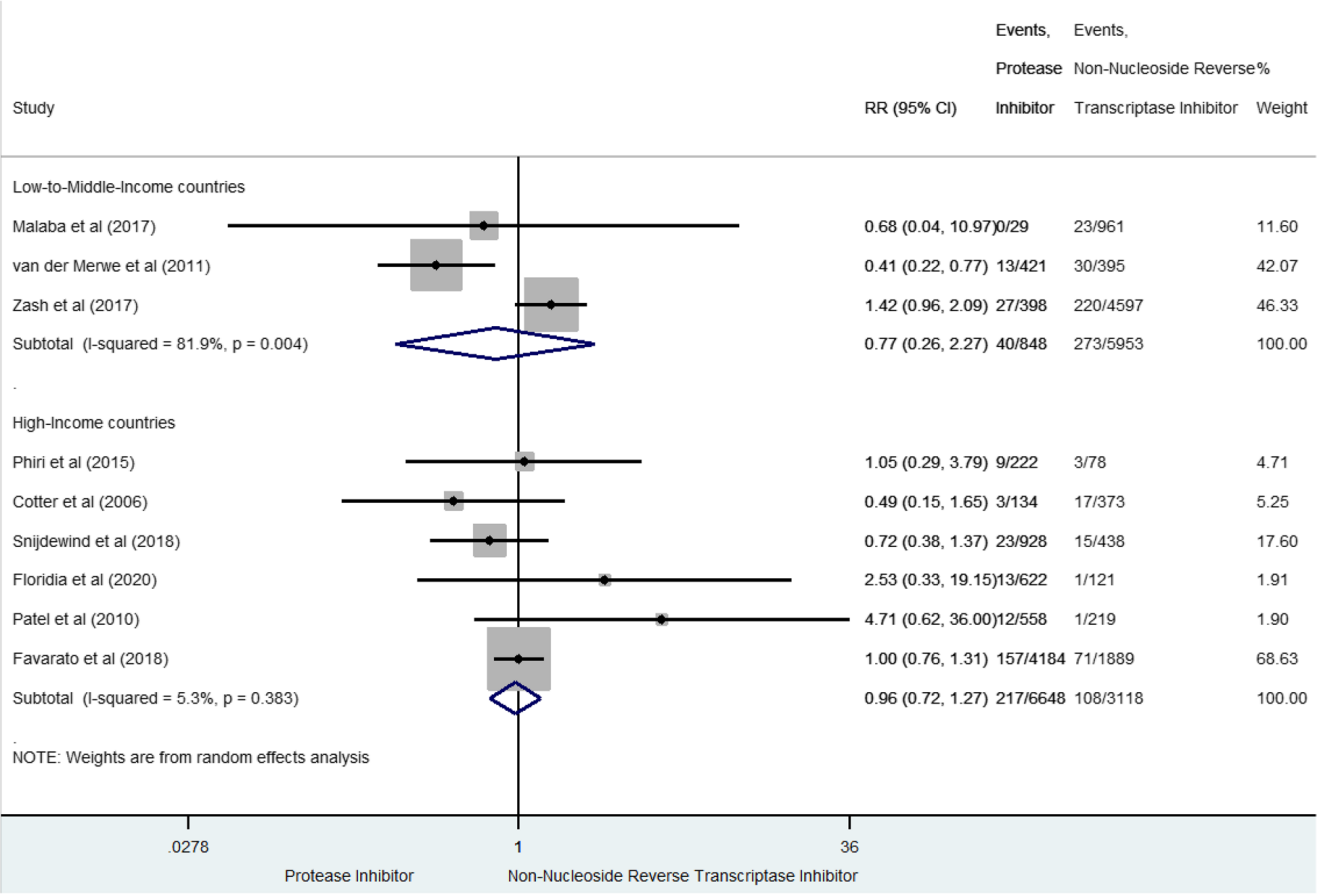
Forest-plot of very preterm-birth risks in pregnant women receiving PI-based compared to NNRTI-based antiretroviral combination

We found a total of 638 VPTB from nine studies (3.9% [3.6–4.1]) [36, 37, 41, 42, 47, 53, 55, 59, 60] (Fig. 3). In LMIC, prenatal exposure to PI-based combination was not significantly associated with VPTB compared to NNRTI-based combination (RR 0.77, 95% CI 0.26–2.27), but with significant between-study heterogeneity (I^2^ = 81.9%, *p* = 0.004). No association was either found in HIC (RR 0.96, 95%CI 0.72–1.27, I^2^ 5.3%, *p* = 0.383). Results were homogeneous when including only studies of high methodological quality [36, 42, 55, 60], reporting a global not significant risk of VPTB (sRR 1.19 [0.89–1.60], I^2^ 15.1% *p* = 0.318).

### Low birth weight

**Fig. 4:**
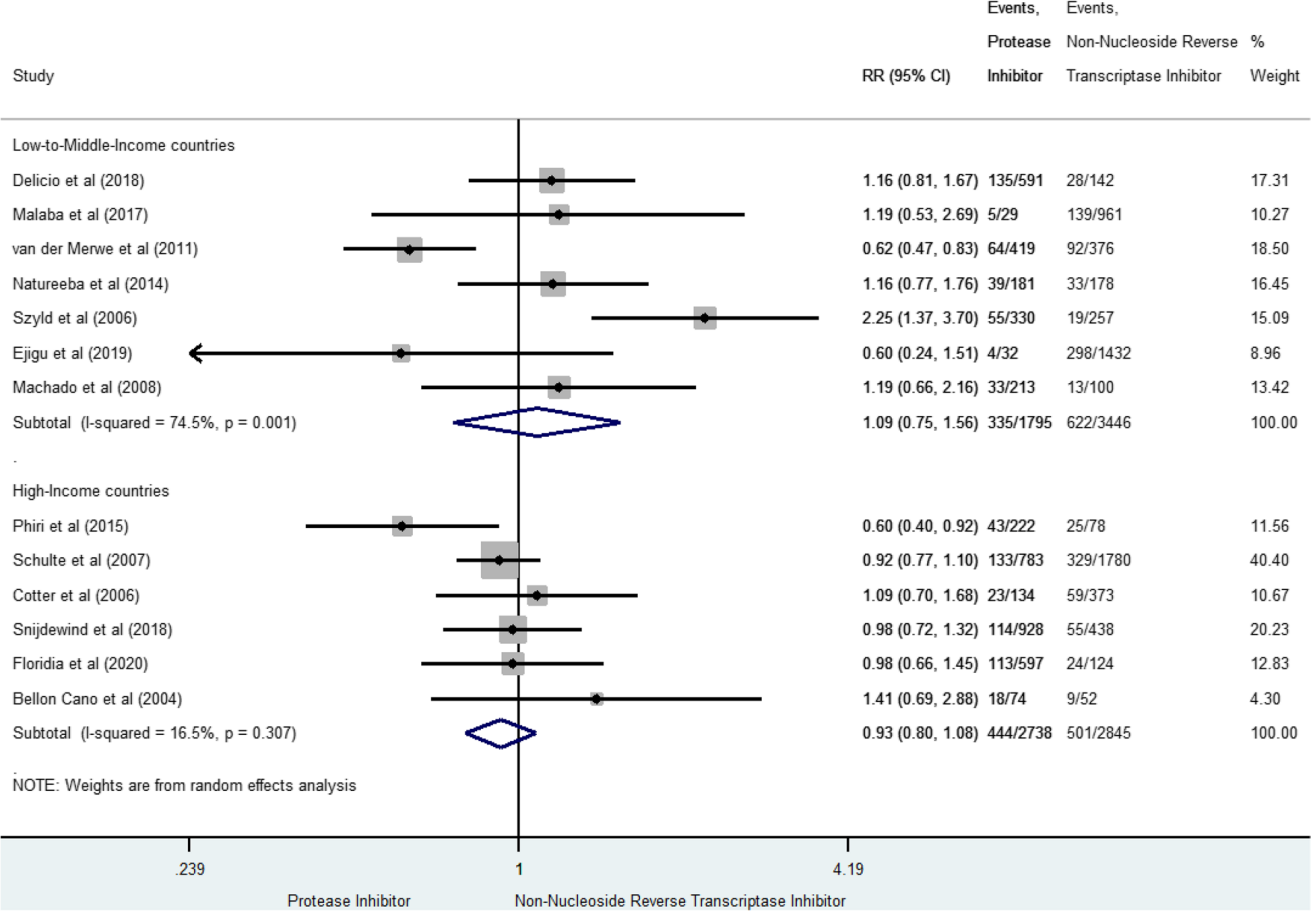
Forest-plot of low-birth-weight risks in pregnant women receiving PI-based compared to NNRTI-based antiretroviral combination

LBW was measured in thirteen studies for 1,902 cases (17.6% [16.9–18.3]) [10, 36, 37, 41, 47, 49, 50, 53, 55–57, 63, 64] (Fig. 4). In LMIC, prenatal exposure to PI-based combination was not significantly associated with LBW compared to NNRTI-based combination (RR 1.09, 95% CI 0.75–1.56), but between-study heterogeneity was significant (I^2^ = 74.5%, *p* < 10^–4^). In HIC, the RR was not significant (RR 0.93, 95%CI 0.80–1.08), and homogeneous (I^2^ 16.5%, *p* = 0.307). Global results were homogeneous when including only studies of high methodological quality [52, 55, 59, 64], reporting a non-significant risk (sRR 1.04 [0.81–1.33], I^2^ 0% *p* = 0.591).

### Very low birth weight

**Fig. 5:**
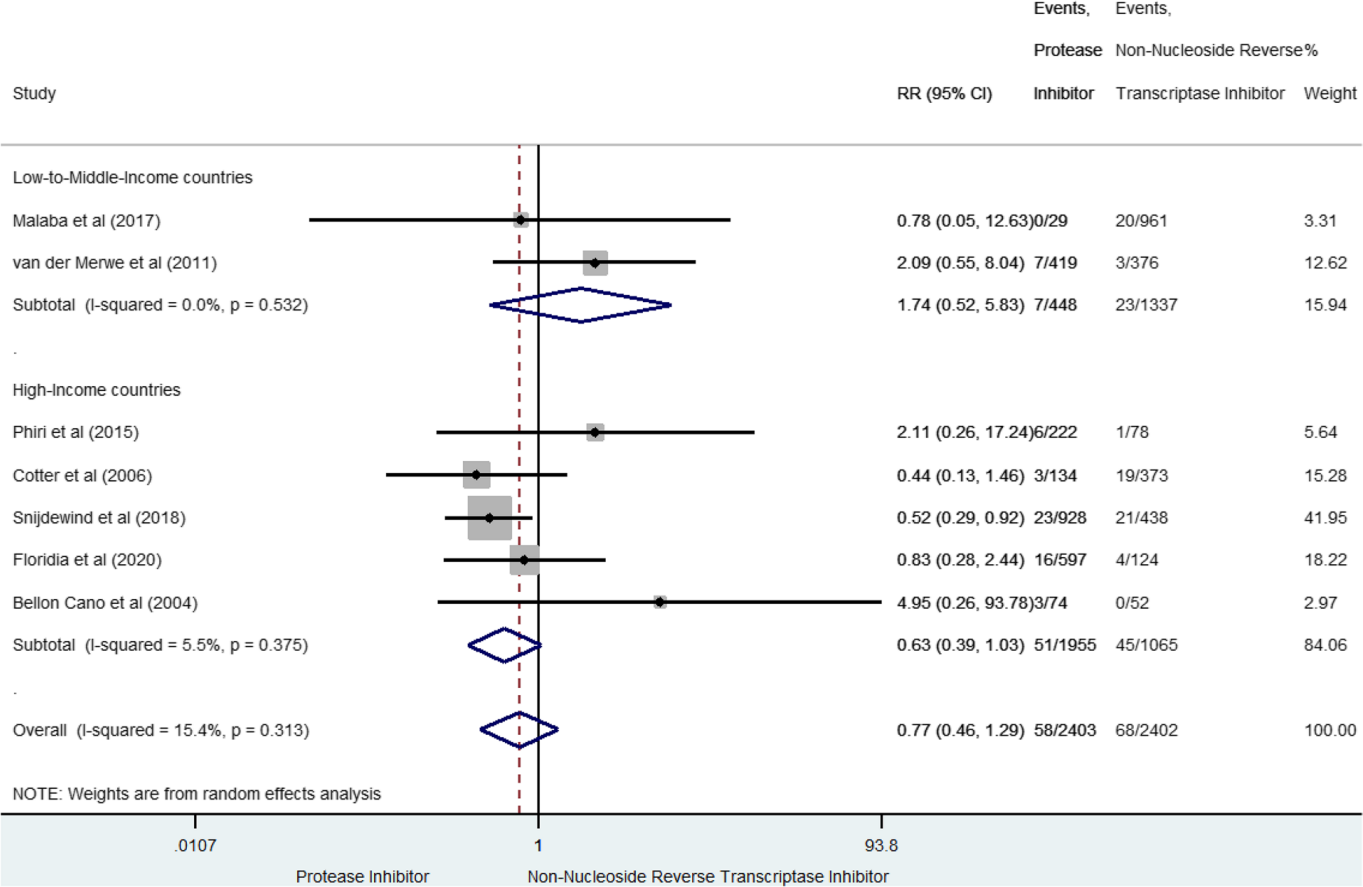
Forest-plot of very low-birth-weight risks in pregnant women receiving PI-based compared to NNRTI-based antiretroviral combination

VLBW was reported in 126 cases across seven studies (2.6% [2.2–3.1]) [36, 37, 41, 47, 53, 55, 63] (Fig. 5). Globally, prenatal exposure to PI-based combination was not significantly associated with VLBW compared to NNRTI-based combination (sRR 0.77, 95% CI 0.46–1.29), and without significant between-study heterogeneity (I^2^ 15.4%, *p* = 0.313). Two studies [36, 55] had a high score of methodological quality and found similar results to those of the main analysis (sRR 0.82 [0.30–2.25], I^2^ 0% *p* = 0.968).

### Small for gestational age

**Fig. 6:**
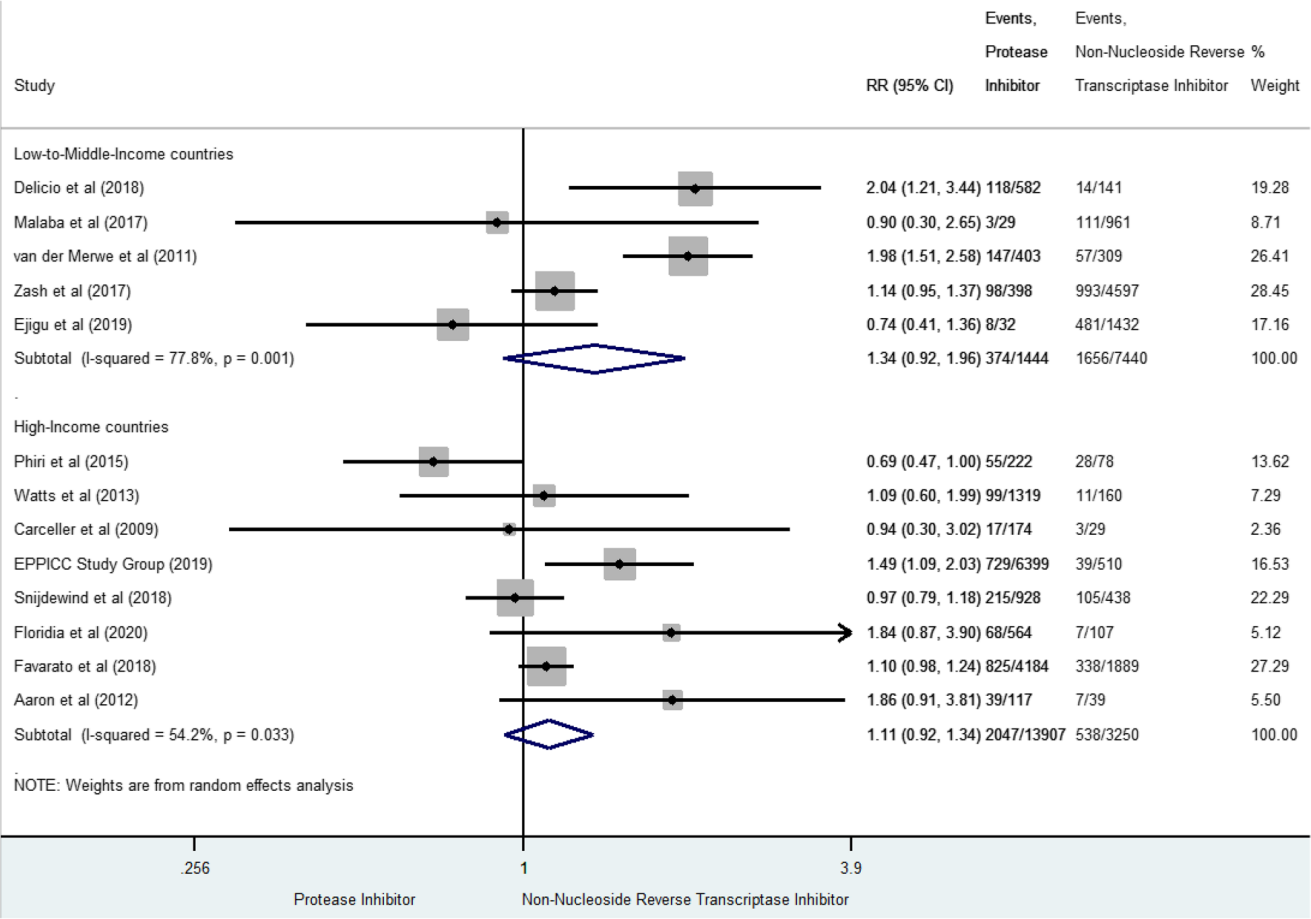
Forest-plot of small-for-gestational-age risks in pregnant women receiving PI-based compared to NNRTI-based antiretroviral combination

The risk of SGA was reported in thirteen studies for 4615 cases (17.7% [17.3–18.2]) [18, 36, 37, 41, 42, 48, 52, 53, 55, 56, 60, 61, 64] (Fig. 6). In LMIC, prenatal exposure to PI-based combination was not significantly associated with SGA compared to NNRTI-based combination but significantly heterogeneous (RR 1.34, 95% CI 0.92–1.96, I^2^ 77.8%, *p* = 0.001). In HIC, we found similar results (RR 1.11, 95%CI 0.92–1.34 and I^2^ 54.2%, *p* = 0.033). When including only studies with high score of quality, we found similar results than those obtained in the main analysis: in LMIC [36, 42, 56, 64] (RR 1.18 [0.80–1.73], I^2^ 56.5% *p* = 0.075) and HIC [55, 60, 61] (RR 1.35 [0.92–1.99], I^2^ 45.5% *p* = 0.160).

### Very small for gestational age

**Fig. 7:**
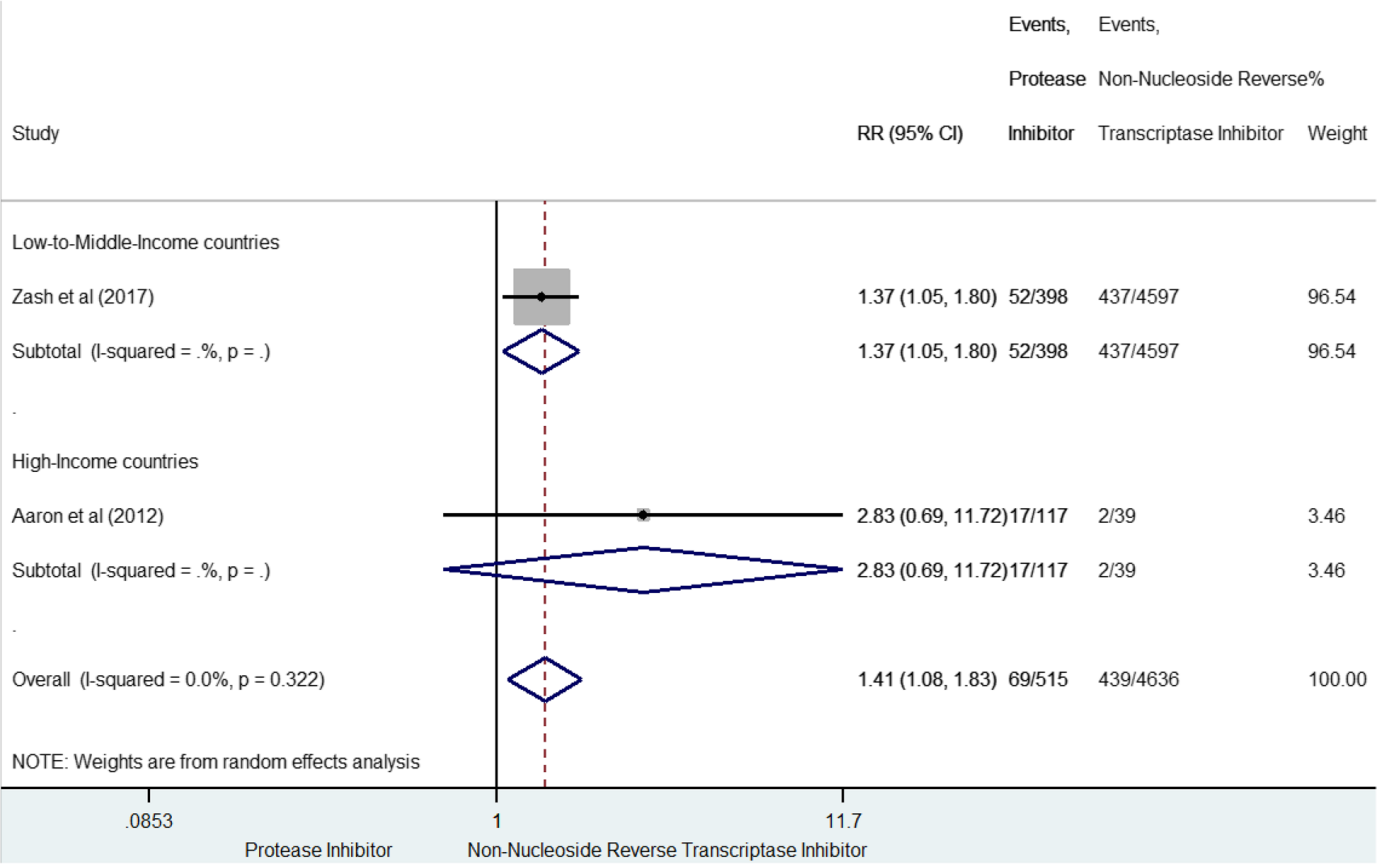
Forest-plot of very small-for-gestational-age risks in pregnant women receiving PI-based compared to NNRTI-based antiretroviral combination

The risk of VSGA, which was reported in two studies for 508 cases (9.9% [9.0–10.7]) [42, 61] (Fig. 7), was globally significantly increased for prenatal exposure to PI-based compared to NNRTI-based combinations (sRR 1.41, 95% CI 1.08–1.84), and consistent between-studies (I^2^ = 0%, *p* = 0.322). Only two studies with a high score of methodological quality contributed to this outcome, which did not allow to evaluate the publication bias.

### Stillbirth

**Fig. 8:**
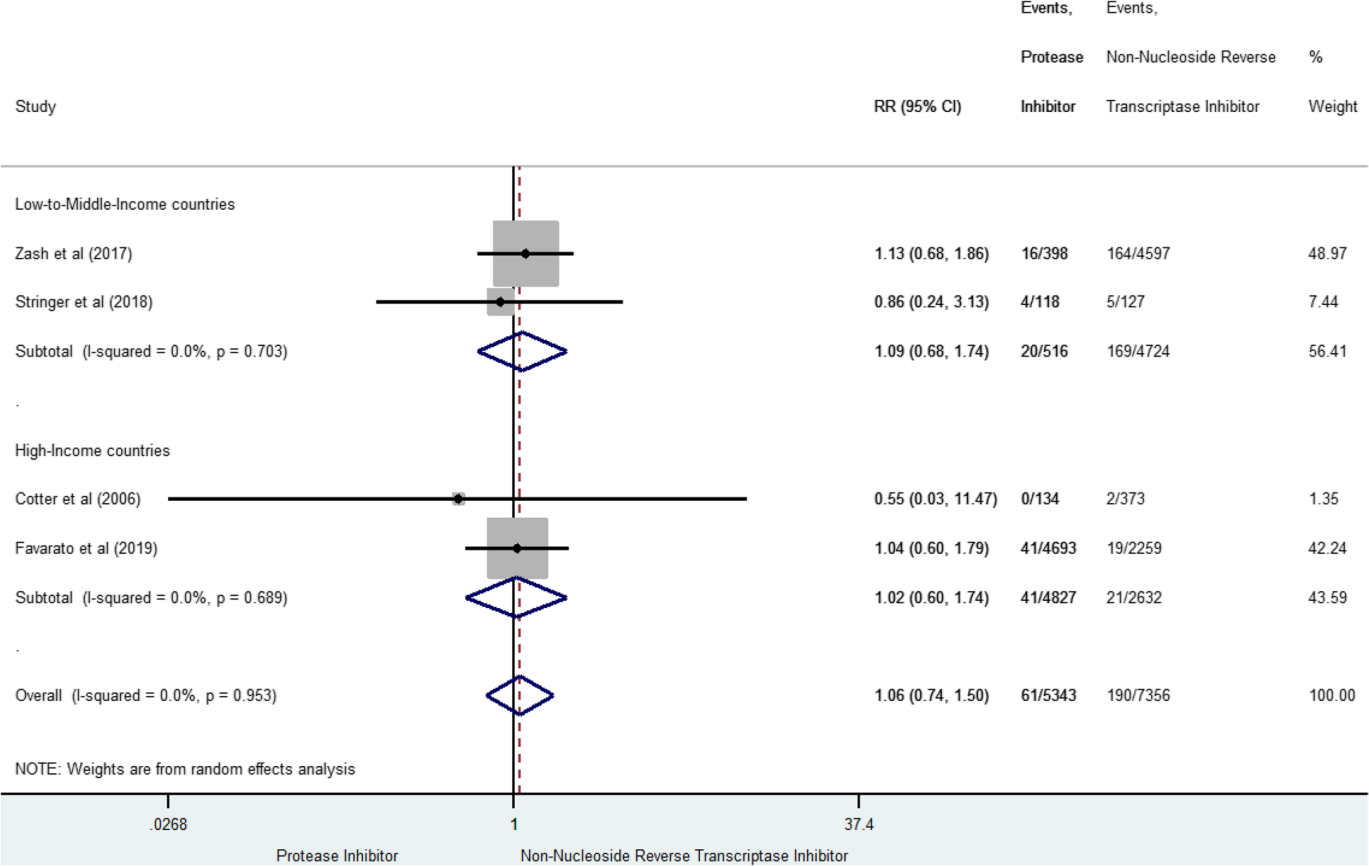
Forest-plot of stillbirth risk in pregnant women receiving PI-based compared to NNRTI-based antiretroviral combination

Stillbirth was reported in four studies for 269 cases (2.0% [1.7–2.2]) [42, 47, 58, 62]. Globally, prenatal exposure to PI-based combination was not significantly associated with stillbirth risk compared to NNRTI-based combination (sRR 1.06, 95% CI 0.74–1.50) (Fig. 8), and consistent between studies (I^2^ = 0%, *p* = 0.953). We found similar results when including only the two studies with high methodological quality [42, 62] (sRR 1.09 [0.75–1.57], I^2^ = 0%, *p* = 0.829).

### Congenital abnormalities

**Fig. 9:**
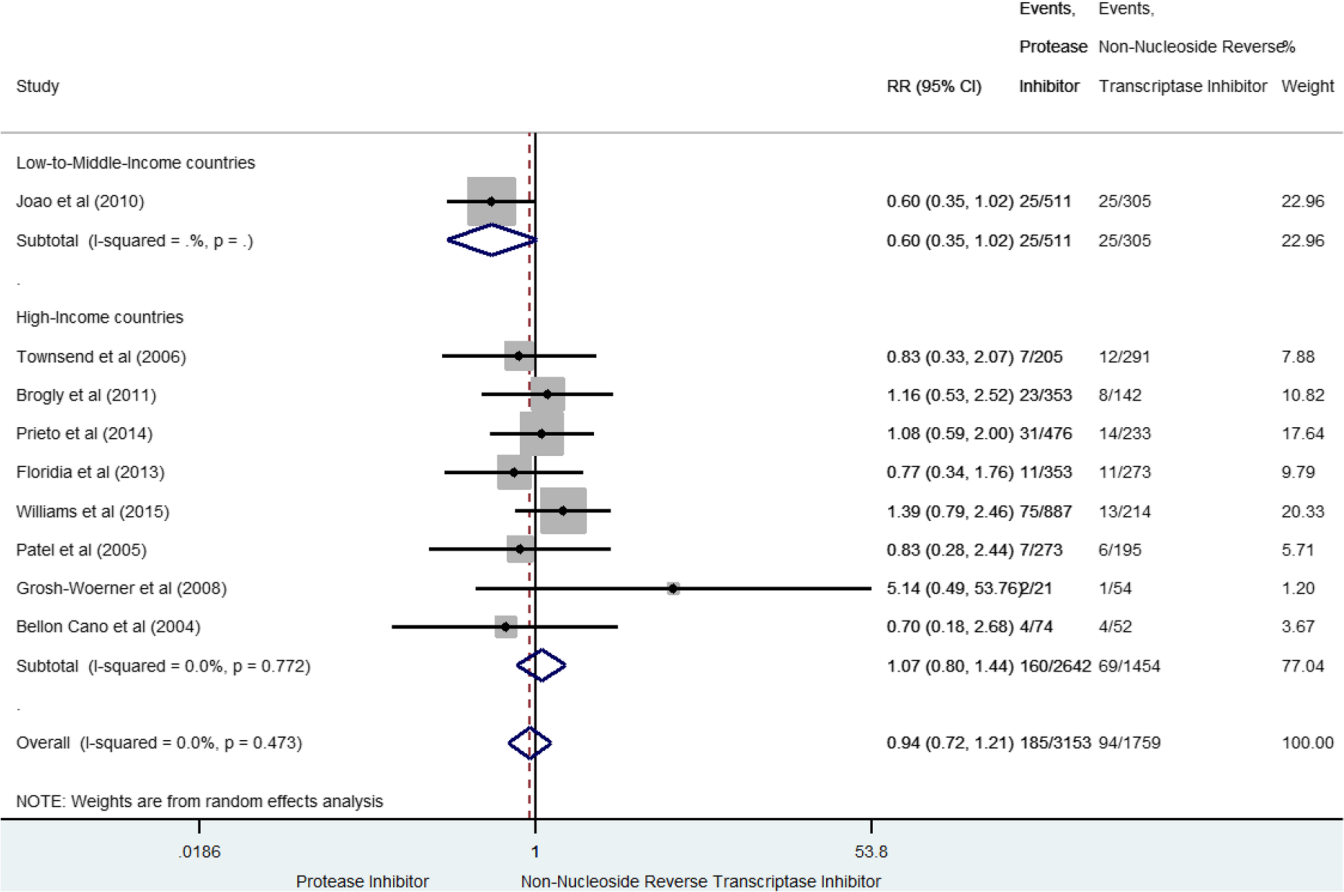
Forest-plot of congenital abnormalities risk in pregnant women receiving PI-based compared to NNRTI-based antiretroviral combination

Congenital abnormalities were reported in nine studies for 279 cases (5.7% [5.0–6.3]) [35, 38–40, 43, 44, 46, 51, 63] (Fig. 9). Globally, prenatal exposure to PI-based combination was not significantly associated with congenital abnormalities compared to NNRTI-based combination (sRR 0.94, 95% CI 0.73–1.21). Between-study heterogeneity was not significant (I^2^ 0%, *p* = 0.473). The three studies [38, 39, 43]] with a high score of methodological quality reported also a non-significant risk (sRR 1.22 [0.84–1.76], I^2^ 0%, *p* = 0.832).

### Spontaneous abortion

Spontaneous abortion was reported in only one study for 50 cases (20.4% [15.4–25.5]) [58]. In this study, prenatal exposure to PI-based combination was not significantly associated with spontaneous abortion compared to NNRTI-based combination (RR 1.26, 95% CI 0.77–2.08). No meta-analysis was conducted.

### Exposure period of antiretroviral combination during pregnancy

We were not able to conduct subgroup analyses by exposure pregnancy periods because only one reported numbers of adverse perinatal outcomes according to the pre-conception period [53], and another one according to early/late pregnancy period (before or after 28 weeks of pregnancy) [41].

## Discussion

While there is an increasing number of pregnant women living with HIV receiving antiretroviral therapy, especially in sub-Saharan Africa, the risk of adverse perinatal outcomes according to antiretroviral combinations exposure remains critical of fully assess. Our meta-analysis specifically assessed these risks associated with maternal PI-based antiretroviral combination still recommended by WHO as second- or third-line therapy, using a head-to-head comparison to NNRTI-based combination, based on a large sample size of pooled studies and originally stratified according to country income.

By pooling the estimates assessed from 32 studies, our meta-analysis comparing the risk of adverse perinatal outcome after prenatal exposure to PI-based compared to NNRTI-based combinations, in both LMIC and HIC countries, provided the following findings: firstly, we did not report any global significant pooled risk consistently for VLBW, stillbirth and congenital abnormalities. Secondly, despite significant heterogeneity between LMIC and HIC studies, there was no significant risk related to PI-ART exposure reported for VPTB, LBW and SGA. Thirdly, we found a global significant increased pooled risk of PI- based regimen for VSGA (+ 41% [8–84]), in two studies. Fourthly, we cannot formally conclude for the global risk of PTB with inconsistent findings according to sensitivity analysis. Lastly, no meta-analysis was conducted for the risk of spontaneous abortion, reported in only one study.

Three other systematic reviews previously published in 2018, 2020, and 2022 reported the effects of PI-based antiretroviral therapy associated with adverse perinatal outcomes [15, 16, 65]. Saleska et al. reported significant higher risk of LBW when only compared to zidovudine (ZDV) monotherapy, but no significant effect compared to NNRTI-based ART [65]. A network meta-analysis of randomized controlled trials conducted by Tsuivila-Matala, also reports that lopinavir/ritonavir (LPV/r) based regimens were associated with a significant higher risk of LBW compared to ZDV monotherapy, but this was not significant compared to NNRTI-based regimens [16]. In 2022, another meta-analysis reported the PI-ART-related adverse perinatal outcomes risks to be significantly higher for SGA (+ 24%,95% CI 8%-43%), and VSGA (+ 40%; 95% CI 9%-81%), but not with PTB or other perinatal outcomes [15].

Based on our results, prenatal exposure to PI-based ART is significantly associated with a increased risk for VSGA, as also recently reported by Cowdell et al. [15]. Some studies have shown that prenatal exposure to PI-based combination was associated with decreased progesterone levels during pregnancy, resulting in elevated estradiol levels [66–68]. Progesterone levels are correlated and estradiol levels are inversely correlated with birth weight. These hormonal changes, induced by prenatal exposure to PI-based combination, may be associated with fetal growth restriction and therefore with a higher VSGA risk [69, 70]. This hypothesis needs to be further investigated to better understand the potential effect of PI-based combination exposure on foetal growth.

In this meta-analysis, some perinatal outcomes (VSGA and spontaneous abortion) are reported in few studies, limiting result interpretation. Spontaneous abortion was investigated in only one study [58], with no meta-analysis conducted for this outcome. For VSGA, one large sample-size study [42] in Botswana, conducted in women who delivered in maternity wards at the national level using a standardized definition of perinatal outcomes, reported an increased risk of VSGA after prenatal exposure to PI-based combination compared to NNRTI-based combination. A standardized definition of gestational age was used in this study, while it is not necessarily the case in others LMIC, providing confidence in pregnancy outcome data quality. Another study [61], in HIC, did not find a significant result of VSGA, but weighed only 3.5% in the overall analysis of this risk. The results obtained for the VSGA risk seem robust thanks to large sample size study and use of standardized definition of perinatal outcomes, but need to be further investigated. Secondly, despite the subgroup analysis stratified between HIC and LMIC, between-study heterogeneity remains significant for most of the perinatal outcomes (PTB, VPTB, LBW and SGA), which may limit the interpretation of our results. This heterogeneity is partly explained by the diversity of methods used to measure perinatal outcomes. We can suppose that estimation of gestational age was more accurate in HIC compared to LMIC, because ultrasound is usually performed at least once during pregnancy in HIC. The heterogeneity for PTB risk can also be explained by one outlier study, excluded in sensitivity analysis in LMIC. The van der Merwe study [41] was conducted in South Africa between 2004 and 2007, and included pregnant women with a median CD4 count of 155 cells/mm^3^. NNRTI exposure was preferred for women with advanced HIV infection or co-infected with tuberculosis. Tuberculosis can be associated with growth restriction and PTB [71, 72]. It can explain outlier data with high prevalence for NNRTI exposure. Without this study, we found a significant increased risk (+ 26%) for PTB associated to prenatal exposure to PI-based combinations, compared to NNRTI-based combinations. This result was also find in the sensitivity analysis conducted only on studies with high scores of methodological quality (+ 20%). Therefore, the result reported in the main analysis appears not robust and heterogeneous. It must be interpreted cautiously, considering the significant association found in the two sensitivity analyses. Moreover, we cannot conduct sensitivity analyses excluding outlier studies for others perinatal outcomes (VPTB, LBW and SGA) due to conflicting results. Indeed, results in the main analysis were heterogeneous, especially in LMIC. However, we found homogeneous and similar results in the sensitivity analyses including only studies with high scores of methodological qualities (VPTB and LBW), showing the robustness of the main analysis results. Last, despite the sensitive analysis conducted on high quality studies, the result of SGA remained heterogeneous but still not significant. As we found a significant increased risk of VSGA, we supposed that no significant risk was reported due to lack of statistical power in the subgroup analyses. Since these two perinatal outcomes are strongly correlated (SGA and VSGA), we suggest to conduct further investigations on the effect of PI-based combination exposure on overall foetal growth.

Our systematic review has some limitations. We could not disentangle effects of HIV exposure also associated with adverse perinatal outcomes [2] from those associated with antiretroviral exposure. The impact of the timing of ART initiation in pregnancy (before/after conception) on perinatal outcomes remains uncertain, as we were not able to conduct the sensitivity analyses due to the lack of information. In our study, we did not specifically explore the effect of NRTI backbone, but it would be relevant to investigate the potential association between PTB and VPTB risks and exposure to ZDV-3TC-LPV/r. Finally, we were not able to investigate the effects on perinatal outcomes according the different PI-based regimen.

Our systematic review has also strengths. Our search was exhaustive thanks to use of several bibliographical databases, abstracts of HIV conference and clinical trials registry. Studies included all available comparative study designs (randomized clinical trials and cohorts) to guarantee the representativeness. No restriction for geographical area and publication date ensured representative results. The use of an appropriate comparator defined as a NNRTI antiretroviral combination allowed us to estimate relative risks, strong indicators of risk. Methodological quality assessment was also performed by two investigators independently. Standardization of data collection and outcome definition raise many challenges in data quality assessment [73]. Our results were detailed according to country-outcome to consider data quality heterogeneity. We conducted sensitivity analyses including only studies with high methodological quality and results were mostly consistent with those of the main analysis. Most of the results obtained in the subgroup analyses were consistent with those obtained in primary analysis, with robust analysis for most of the outcomes, except for PTB.

## Conclusion

Our study did not show a higher risk for most of the adverse perinatal outcomes after prenatal exposure to PI-based combination compared to NNRTI-based combination. However, our review suggests a significant increased risk of VSGA, similarly reported in another recent review [15]. The risk of PTB initially reported is not clearly demonstrated [10, 18, 30, 31, 36, 37, 41, 45, 48, 49, 52, 53, 55–59, 63, 64], with significant between-studies heterogeneity. Therefore, this result should be interpreted with caution. Our results should be considered to inform clinical guidelines, with appropriate messaging regarding the PI benefit-risk balance in pregnant women and those of childbearing potential living with HIV to improve their perinatal outcomes.

## Data Availability

All data generated or analyzed during this study are included in this published article.

## Abbreviations

ALT: Alanine aminotransferase
AST: Aspartame aminotransferase
ATHENA: AIDS Therapy Evaluation in the Netherlands
CI95: Confidence interval 95
ECS: European Collaborative Study
HAART: Highly Active Antiretroviral Therapy
HIV: Human Immunodeficiency Virus
IPMTCT: Integrated Prevention of Mother To Child Transmission
EFV: Efavirenz
EPPICC: European Pregnancy and Paediatric HIV Cohort Collaboration
LBW: Low birth weight
LPV/r: Lopinavir/ritonavir
MTCT: Mother to Child Transmission
NA: Not available
NSHPC: National Study of HIV in Pregnancy and Childhood
ND: Not define
NISDI: NICHD (National Institute of Child Health & Human Development) International Site Development Initiative
NNRTI: Non-nucleoside reverse transcriptase inhibitor
non PI: Considered here like NNRTI
NVP: Nevirapine
PI: Protease inhibitor
PSD: Pediatric Spectrum of HIV Disease
RR: Relative Risks
SGA: Small for gestational age
SMARTT: Surveillance Monitoring of ART Toxicities, the French ANRS (National Research Agency on HIV and hepatitis)
TS: Trimethoprim-sulfamethoxazole
VLBW: Very low birth weight
VPT: Very preterm birth
VSGA: Very small for gestational age
WHO: World Health Organisation
